# Defining Natural History: Assessment of the Ability of College Students to Aid in Characterizing Clinical Progression of Niemann-Pick Disease, Type C

**DOI:** 10.1371/journal.pone.0023666

**Published:** 2011-10-03

**Authors:** Jenny Shin, Katrina Epperson, Nicole M. Yanjanin, Jennifer Albus, Laura Borgenheimer, Natalie Bott, Erin Brennan, Daniel Castellanos, Melissa Cheng, Michael Clark, Margaret Devany, Courtney Ensslin, Nina Farivari, Shanik Fernando, Lauren Gabriel, Rani Gallardo, Moriah Castleman, Olimpia Gutierrez, Allison Herschel, Sarah Hodge, Anne Horst, Mary Howard, Evan James, Lindsey Jones, Mary Kearns, Mary Kelly, Christine Kim, Kinzie Kiser, Gregory Klazura, Chris Knoedler, Emily Kolbus, Lauren Lange, Joan Lee, Eileena Li, Wei Lu, Andrew Luttrell, Emily Ly, Katherine McKeough, Brianna McSorley, Catherine Miller, Sean Mitchell, Abbey Moon, Kevin Moser, Shane O'Brien, Paula Olivieri, Aaron Patzwahl, Marie Pereira, Craig Pymento, Erin Ramelb, Bryce Ramos, Teresa Raya, Stephen Riney, Geoff Roberts, Mark Robertshaw, Frannie Rudolf, Samuel Rund, Stephanie Sansone, Lindsay Schwartz, Ryan Shay, Edwin Siu, Timothy Spear, Catherine Tan, Marisa Truong, Mairaj Uddin, Jennifer VanTrieste, Omar Veloz, Elizabeth White, Forbes D. Porter, Kasturi Haldar

**Affiliations:** 1 Center for Rare and Neglected Diseases, University of Notre Dame, Notre Dame, Indiana, United States of America; 2 Program in Developmental Endocrinology and Genetics, NICHD, National Institutes of Health, DHHS, Bethesda, Maryland, United States of America; 3 NPC Consortium for Community-Based Assessment of Patient Records, Cape Town, South Africa; Charité Universitätsmedizin Berlin, NeuroCure Clinical Research Center, Germany

## Abstract

Niemann-Pick Disease, type C (NPC) is a fatal, neurodegenerative, lysosomal storage disorder. It is a rare disease with broad phenotypic spectrum and variable age of onset. These issues make it difficult to develop a universally accepted clinical outcome measure to assess urgently needed therapies. To this end, clinical investigators have defined emerging, disease severity scales. The average time from initial symptom to diagnosis is approximately 4 years. Further, some patients may not travel to specialized clinical centers even after diagnosis. We were therefore interested in investigating whether appropriately trained, community-based assessment of patient records could assist in defining disease progression using clinical severity scores. In this study we evolved a secure, step wise process to show that pre-existing medical records may be correctly assessed by non-clinical practitioners trained to quantify disease progression. Sixty-four undergraduate students at the University of Notre Dame were expertly trained in clinical disease assessment and recognition of major and minor symptoms of NPC. Seven clinical records, randomly selected from a total of thirty seven used to establish a leading clinical severity scale, were correctly assessed to show expected characteristics of linear disease progression. Student assessment of two new records donated by NPC families to our study also revealed linear progression of disease, but both showed accelerated disease progression, relative to the current severity scale, especially at the later stages. Together, these data suggest that college students may be trained in assessment of patient records, and thus provide insight into the natural history of a disease.

## Introduction

Niemann-Pick Disease, type C (NPC) is a lysosomal, cholesterol storage disorder [1]. It is an autosomal, recessive, rare disease with a prevalence in the range of 1∶120,000 to 150,000 [2]. Although the earliest manifestations are splenomegaly and hepatomegaly, clinically, it presents as a broad spectrum of neurological problems. This in conjunction with highly variable age of onset (ranging from fetal to adult) makes it difficult to diagnose [3, also reviewed in 2]. Indeed, the average time from initial symptom to diagnosis is approximately 4 years. Even after diagnosis, which comprises of a skin biopsy to obtain fibroblasts to demonstrate accumulation of unesterified cholesterol or genetic testing, there is need for a universally accepted clinical outcome measure for testing urgently needed new therapeutics as well as disease management. Clinical investigators have defined emerging, disease severity scales [4], [5], the most comprehensive of which was recently reported by Yanjanin et al., 2010 [6], as part of a natural history study being conducted at the National Institutes of Health (NIH).

However, since the incidence of NPC is low, the number of patients seen in a clinical center is limited. Many patients will not travel to specialized clinical centers (even a national center like the NIH) after diagnosis, because of the paucity of therapies, lack of information about programs, as well as significant logistical issues. In addition to enrolling 18 patients on the current study, Yanjanin et al. [6] were also able to utilize 19 clinical records archived at the NIH, and this led to the suggestion that assessment of patient records donated by families including records of patients who have died from the disease, may be used to rapidly strengthen emerging clinical severity scales.

For a rare disease like NPC, scales derived from a larger set of clinical records would represent a significant advance in capturing a standardized clinical outcome measure. However due to limited resources associated with rare disease research, only a number of physicians specialize in NPC, suggesting that community-based assessment of clinical records by trained volunteers could greatly facilitate generation of disease severity scales and other clinical tools. As a first step towards this goal we undertook to develop a scalable model in which pre-existing medical records may be correctly assessed by non clinical practitioners trained to quantify disease progression and assemble succinct clinical summaries. If successful, this model could be implemented to assist in the collection of natural history data for other rare diseases.

## Methods

### Ethics statement

The study was an extension of a Natural History study performed at the National Institutes of Health in Bethesda, Maryland. It was approved by the University of Notre Dame Institutional Review Board (09-174), as well as the Eunice Kennedy Shriver National Institute of Child Health and Human Development Institutional Review Board (06-CH-0186, *NCT00344331*). Consent and if appropriate assent, were obtained from guardians and patients. All students received HIPAA training in class, prior to reviewing private patient data.

### Participants and procedures

The study was developed to ascertain whether non-clinical practitioners who were trained over a few weeks to recognize NPC clinical disease, could extract clinical severity scores from patient records. The clinical severity scale of Yanjanin et al. 2010 [6] was adopted. This scale has been validated with data from 37 patient records. 18 of these records were from a cross sectional study of current NPC patients. An additional 19 were obtained from longitudinal chart review of a ‘historic’ cohort at the NIH, and thus the scale is expected to be useful as a long-term outcome measure for biomarkers and therapeutic trials. The scale utilizes nine major and eight minor clinical symptomatic domains, with a Likert-like scale to assign scores of 0-5 and 0-2 in each domain-type respectively. The total scores can range from 0 to 61 and higher scores indicate increased disease burden.

Training of students was done in an in-class room setting because this is an easily scalable format. The class, entitled Developing Health Networks in Rare and Neglected Diseases was developed by the Center for Rare and Neglected Diseases (CRND) at the University of Notre Dame in collaboration with the Eunice Kennedy Shriver National Institute of Child Health and Human Development (NICHD) and consultation with the Center for Social Concerns at the University of Notre Dame, Riley Children's Hospital, the National Niemann Pick Disease Foundation (NNPDF), the Ara Parseghian Medical Research Foundation (APMRF) and the Niemann-Pick Disease Group (UK).

5-30 students were enrolled per class for four classes in Spring 2009, Fall 2009, Spring 2010 and Spring 2011. Registration required the instructor's permission (the class was restricted to upper level undergraduate students). For the initial class in Spring 2009 two graduate students were also allowed to enroll to determine whether the course was better suited to post graduate rather than undergraduate students A total of sixty four undergraduates and two graduate students were enrolled over the indicated semesters. The class met once a week for 3 contact hours.

All students registered for the course indicated no prior experience in clinical research. Thus, for all students, this class represented the first exposure to critical concepts pertinent for developing therapies for rare and neglected diseases in general and specifically Niemann-Pick type C. Students researched multiple NPC family narratives publicly available via the internet, to understand the socioeconomic and cultural impact of NPC disease. Students were then instructed on NPC diagnosis, symptoms and clinical history through a combination of didactic lectures and reading of clinical research papers, as well as discussions with clinical practitioners and researchers in NPC disease (http://nd.edu/~crnd/CTSSeriesAnnouncements.html).

**Figure 1:**
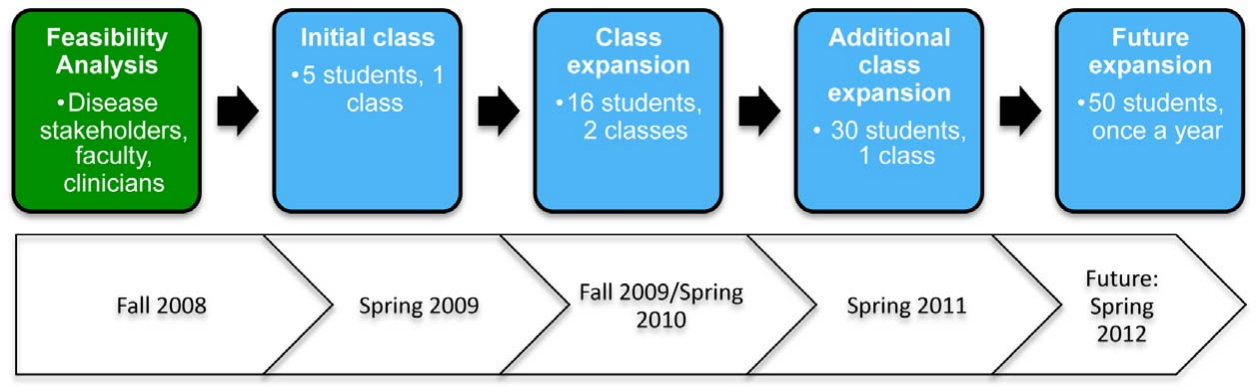
Overall work flow undertaken to assess the ability of college students to assist in characterizing clinical progression of Niemann Pick Type C. The project began in Fall 2008 with discussions with the Center for Social Concerns at the University of Notre Dame, Riley Children's Hospital, Indianapolis, the National Niemann Pick Disease Foundation (NNPDF), the Ara Parseghian Medical Research Foundation (APMRF) and the Niemann-Pick Disease Group (UK), clinical practitioners at Eunice Kennedy Shriver National Institute of Child Health and Human Development (NICHD) and faculty and program staff the Center for Rare and Neglected Diseases (CRND) at the University of Notre Dame.

In Spring 2009, five students (3 undergraduate and 2 graduate students) were assigned a single clinical history previously assessed by Yanjanin et al., 2010 [6]. In Fall and Spring 2010, the class size was expanded to 16 students and an additional three clinical histories from Yanjanin et al. [6] were also assessed by students working in teams of four. In Spring 2011, each of these four clinical histories as well as three additional histories taken from Yanjanin et al., 2010 [6] were assessed in groups of six students scoring in pairs, creating triplicate values for each case. This was done to directly determine the variability in disease severity assessments ascribed to a medical history, when conducted by a pairs of students in the same class environment. In Spring 2011, we also tested the variability in disease severity assessments across 30 students when each student independently assessed disease severity for a new case directly donated to our study. In all semesters, a single clinical visit was first assessed in class to confirm that the logistics of scoring was uniformly understood by the group. Students then scored the entire clinical history as a take home exercise to generate individual disease severity curves. In Spring 2009, Fall 2009 and Spring 2010, the disease severity curves were subsequently reviewed and finalized in consultation with clinical experts pediatric nurse practitioner Nicole M. Yanjanin and physician-scientist Forbes D. Porter from the intramural research program of Eugene Kennedy Shriver National Institute of Child Health and Human Development (NICHD). The indicated disease severity curves incorporated the concept of the ‘carry-over’ effect which is based on the principle that increased score for a symptomatic domain in a prior visit is ‘carried’ through to a subsequent clinical visit in absence of any additional information pertaining to the domain. Thus if a patient is wheel chair dependent on one visit, this is assumed to be true in a subsequent visit, unless specified otherwise. In Spring 2011, the ‘carry-over’ effect was discussed prior to the visits for Yanjanin and Porter in order to incorporate it as a standard feature of ‘in class’ training. The overall work flow is summarized in Figure 1.

NPC families were made aware of the study at the 2009 and 2010 NNPDF family meetings in Seattle, Washington and Toronto, Canada, respectively. Consenting families submitted records through NNPDF to the NIHCD team. An amendment was made to the existing protocol, which then allowed for the records to be forwarded from the NIHCD to the CRND at the University of Notre Dame.

## Results

Early discussions (in Fall 2008) with major stakeholders in the NPC disease community including, foundations, clinical practitioners and families, led to the concept that direct interactions between students and patient groups may fail to protect vulnerabilities of both groups. However, assessment of disease from clinical records by appropriately trained students and collaboration with clinical practitioners was likely to offer a secure process of engagement, training and research.

Comprehensive training of students in clinical NPC disease manifestation, diagnosis and management and requisite medical terminology required 4 weeks of a 12 week semester, with three hours of in-class time and three to six hours of out-of-class time per week. Followed by one, three hour in-class training session focused on a single clinical visit from one record, all assessments were carried out independently by students as part of ‘take-home’ exercises. The first specific product generated by the class was a list of medical terminology used to describe major and minor symptomatic domains (Table 1). This assisted understanding and recognition of symptoms by the students. This list was additionally expanded to include clinical terms encountered in patient histories received over four semesters and has evolved as a key, starting reference tool (Table S1) in assessing new clinical records.

**Table 1:** NP-C terms students *must* know.

**Ambulation**	To walk from place to place; move about.
**Auditory Brainstem Response**	An electrical output in response to sound, a response to a complex signal.
**Biomarkers**	A single characteristic that is indicative of a disease.
**Cerebellar Ataxia**	Gross lack of coordination
**Cognition**	The mental process of knowing, including aspects such as awareness, perception, reasoning, and judgment.
**Cross-sectional**	Taken only once
**Dysarthria**	Motor speech disorder
**Dysmetria**	Lack of coordination of movement typified by the undershoot and/or overshoot of intended position with the hand, arm, leg, or eye. It is sometimes described as an inability to judge distance or scale.
**Dysphagia**	Difficulty swallowing
**Dystonia**	Sustained muscle contractions cause twisting and repetitive movements or abnormal postures.
**Fine Motor Skills**	Coordination of small muscle movements
**Gelastic Cataplexy**	Loosening of the muscle, dropping of the jaw during extreme emotion, such as laughter.
**Hyperlipidemia, Type IV**	A not uncommon inherited metabolic disorder that is characterized by increased blood levels of the triglyceride form of fat that makes up very low-density lipids (VLDL). Abnormally high blood levels of triglycerides or cholesterol may be the result of poor dietary habits, genetic causes, or other metabolic disorders or a side effect of certain drugs. Also known as:
	Carbohydrate-Induced Hyperlipemia
	Hypercholesterolemia, Type IV
	Hyperlipidemia IV
	Hyperprebeta-Lipoproteinemia
	Hypertriglyceridemia, Endogenous
**Hyperreflexia**	Overactive reflex
**Incontinence**	The inability of organs to restrain the natural evacuations, so that the discharges are involuntary; as, incontinence of urine. The involuntary discharge of urine or feces.
**Likert-like scale**	Graded self evaluation
**Longitudinal**	taken over time, multiple data points from expression
**Narcolepsy**	Chronic sleep disorder characterized by overwhelming daytime drowsiness and sudden attacks of sleep.
**Ophthalmology:**	*Distance visual activity (DVA):* Acuteness or clearness of vision, especially form vision, which is dependent on the sharpness of the retinal focus within the eye and the sensitivity of the interpretative faculty of the brain.
	*Extra-ocular movement (EOM):* Can often identify abnormalities in individual muscles or in particular cranial nerves (oculomotor, trochlear, or abducens) in their course from the brainstem to the orbit, in the brainstem nuclei, or finally, in the higher-order centers and pathways in the cortex and brainstem that control eye movements; an example of EOM is saccades.
	*Systemic lupus erythematosus (SLE):* A chronic, relapsing autoimmune disease that may affect skin, joints, and internal organs. The most common ocular finding is the presence of cotton wool spots, retinal hemorrhages, and papilledema. Other symptoms such as lid swelling, exophthalmus, and fluctuating weakness of extraocular muscles may occur.
**Ophthalmoplegia (horizontal and vertical)**	A paralysis or weakness of one or more of the muscles that control eye movement. Eyes don't move togetherà double vision.
**Psychotic Episodes**	Hallucinating, altered reality
**Saccades**	Fast movement of an eye, head or other part of an animal's body or device; eye saccades are quick, simultaneous movements of both eyes in the same direction; serve as a mechanism for fixation, rapid eye movement and the fast phase of optokinetic nystagmus (a type of involuntary eye movement).
**Seizures**	A sudden episode of transient neurologic symptoms such as involuntary muscle movements, sensory disturbances and altered consciousness.
**Supranuclear Vertical Gaze Palsy**	Cannot look upwards, impaired upward gaze
**Xanthoma**	A skin condition in which fat builds up under the surface of the skin; common, particularly among older adults and persons with high blood lipids.
**Also need to know:**	Behavior, eye movement, hearing, memory, psychiatric, speech, swallowing, respiratory problems

### Scoring and disease severity curve generated from NPC patient records obtained from Yanjanin et al., 2010 [6]

**Figure 2:**
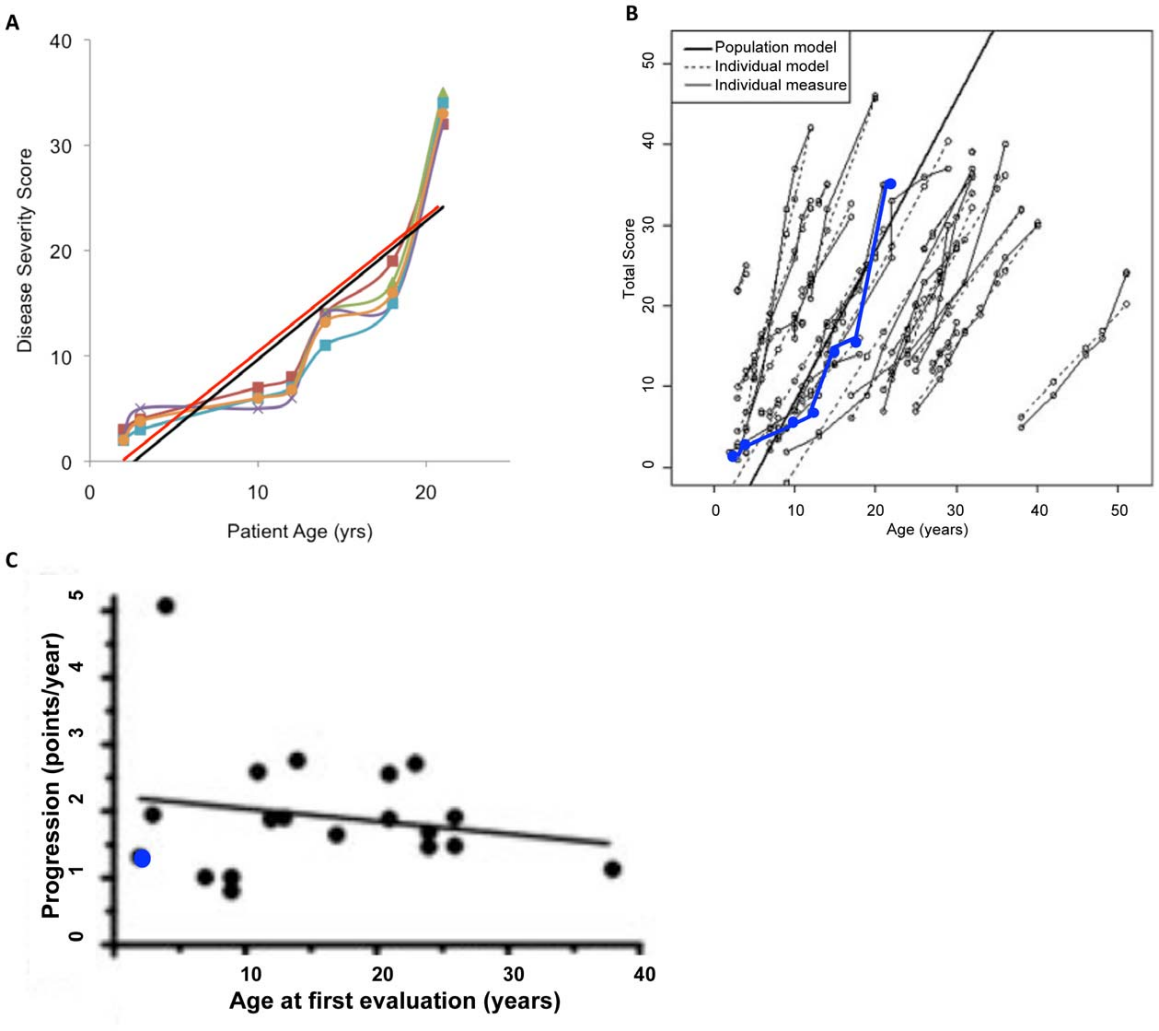
Initial assessment of one NP-C patient medical record by five students in Spring 2009. A. Five students independently scored one NP-C patient's medical record, The disease severity curves were plotted as shown. The correlation among their evaluation is 0.76. The best fit line is shown in black line with a slope of 1.315. In red is the best fit line (with a slope of 1.3 and a correlation of 0.90) obtained by Yanjanin et al. 2010. B. The average of five student scores (show in blue) was overlaid on a summary of disease severity curves obtained by Yanjanin et al 2010 (obtained by combining their historical and current cohorts). C. The progression slope shown in panels A and B was determined by linear regression and overlaid (blue dot) onto the progression slope created by Yanjanin et al. 2010.

The first class in Spring of 2009 was limited to five students, three senior undergraduate students who were also Science majors as well as two graduate students in Biological Sciences. The group assessed one medical history. The small group size and close supervision was deemed appropriate since this type of class on clinical research had not previously been offered for undergraduate students. Five independent disease severity curves were extracted from a single clinical history. As shown in Figure 2A, there was close correspondence between student assessments. For any given symptomatic domain the differences in scores assigned were no greater than 1 (not shown). The indicated disease severity curves incorporated of the concept of ‘carry-over’ (see Methods) The average curve and mean progression score were overlayed on the data of Yanjanin et al. 2010 [6] (Fig. 2B and C). The rate of change for this patient, 1.315 closely corresponds to 1.3 previously reported by Yanjanin et al., 2010 [6].

These data tentatively suggested that upper level undergraduate college students could be trained to recognize disease domains in clinical records and compute disease severity scores. Our impression was that there was no difference in the work product of undergraduate and graduate students. This justified opening subsequent class enrollment to undergraduate juniors and seniors who were declared as Premedical (also referred to as Pre-Professional) and/or majors in Biological Sciences, Chemistry or Biochemistry, and thus an expansion in class size.

**Figure 3:**
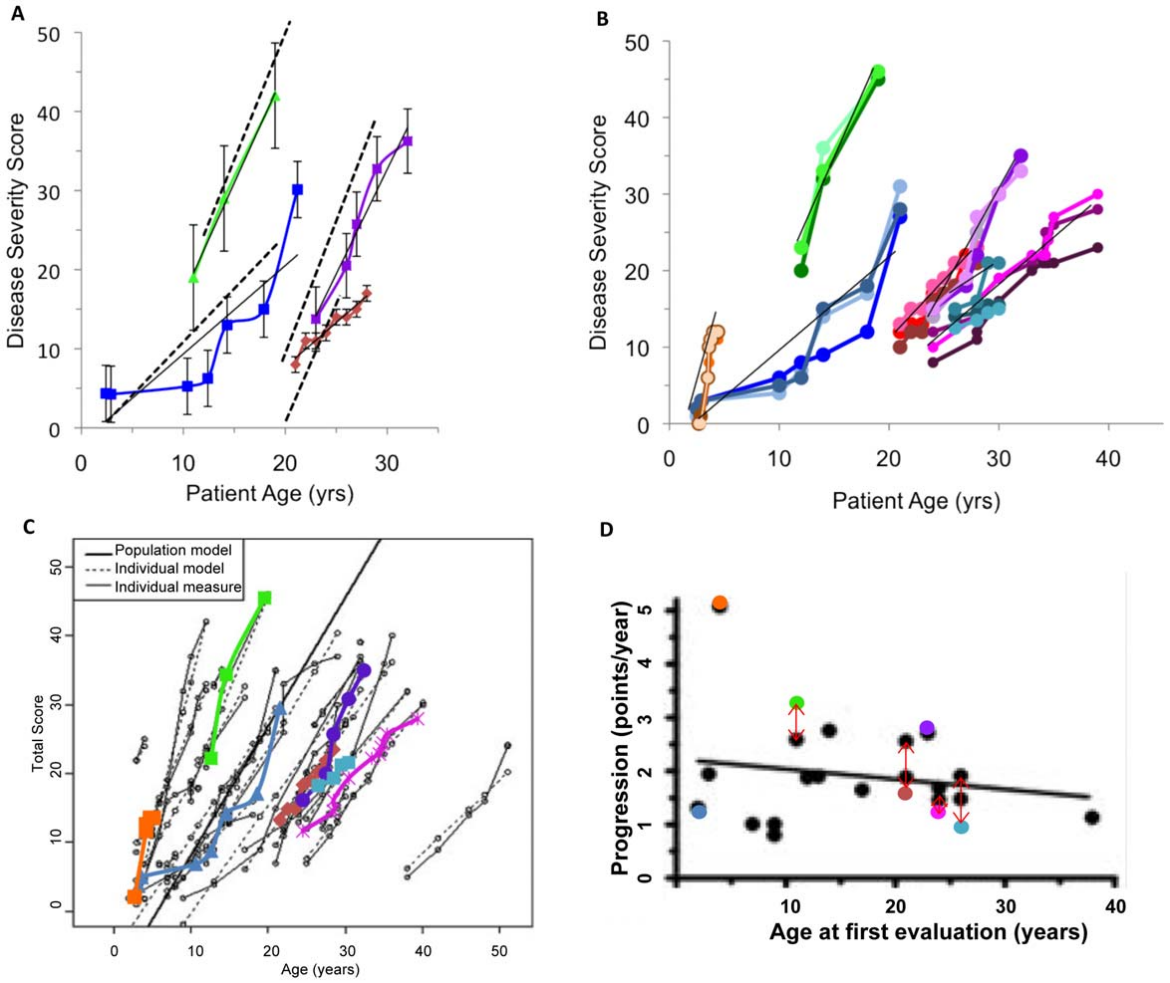
Assessment of four NP-C patient medical records by expanded class sizes. A. Disease severity curves generated by two different classes of sixteen students in Fall 2009 and Spring 2010. From each class groups of three to five students evaluated four NP-C patient's medical records obtained from Yanjanin et al. 2010. Each group evaluated one patient record. Error bars indicate scoring variation between the two classes. Linear best fit shown as solid black line for each record. Hatched line indicates best fit obtained by Yanjanin et al 2010. Color code: case 1, blue; case 2, red; case 3, purple; case 4 green; B. Disease severity curves generated by thirty students in Spring 2011. Five groups of six students scored the four NP-C medical records from panel A as well as three additional records. Scoring was done by pairs of students and triplicate scores were generated for each medical record as shown. Color code as indicated in panel A as well as case 5, magenta; case 6 orange; case 7, teal. Triplicate curves associated with each case, where each curve indicated pair wise scoring. C. The average of curves shown in panel B overlaid a summary of disease severity curves described in Yanjanin et al. 2010. D. The progression slope shown in panel C was determined by linear regression and overlaid onto the progression slope created by Yanjanin et al. 2010. Red arrows indicate extent of displacement of progression obtained from student assessments. Colors as indicated for panel C. When students' progression values were used, the progression slope was found to be 1.907 +/− 0.2374. The original progression slope calculated by Yanjanin et al. 1.923 +/− 0.2200.

With a larger class, we adopted group-wise assessment of clinical records, to minimize operator variability. Thus in Fall 2009 and Spring 2010, when the class sizes were expanded to 14-16, four groups of three to five students were assigned the same clinical record assessed by the initial class as well as three additional medical histories from Yanjanin et al. 2010 [6], for assessment. As shown in Fig. 3A, for a given case, the variation to the mean was comparable across data points in disease progression curves. These data suggested that over two semesters, junior and senior undergraduate students with basic (200 level) background course work in life sciences, could be sufficiently trained in recognition and quantification of disease domains and work in groups of 3–5 to generate consistent disease progression curves from clinical histories.

Since our long term goal is to maximize the capacity of student assessment, in the subsequent semester we increased the class enrollment from 16 to 30. We also investigated the assessment of records by pairs of students. A single case was assigned to three pairs of students within a ‘group’ to rigorously assess variation between pairs within a single class. Of the seven assigned cases, four had been reviewed by the prior classes. The slopes of the disease severity scales in Spring 2011 (Fig. 3B) were comparable to those seen in prior semesters (Fig. 3A), with an average of ∼15% variation in progression measured a year apart. In the remainder, the variation along disease severity curve for one case [6] was indeed low (Fig. 3B). This case was not reported in Yanjanin et al. 2010 [6]. However it was partially scored (one visit) before being transferred to the study and is therefore included. In two records (numbered cases 7 and 5) we noticed a significant variation across the triplicates pairs (Fig. 3B; see also Fig. S1). Discussion with the class suggested that for case 7, fine motor (2-4) and cognition (1-4) were the areas of greatest variance. Importantly at one age (29), one group scored consistently higher than the other group in fine motor skills (2–4 variation over the group), cognition (1–4), and speech (the higher scoring group scored speech 1 pt higher than all other individuals (1–2) (Figure S2). In case 5, the discrepancies in scoring occurred in cognition (1-3) and swallow (3–5) (not shown): Even for case 6, where there was little variation in overall scoring (Fig. 3B and Fig. S1), there were scoring discrepancies between cognition and memory. 1 pair consistently scored cognition 1 and memory 3. 2 other pairs scored cognition 3 and memory 0 (not shown). Another problem with case 7 was that it contained data from just four visits and the main source of error appears to be from visit 3, when pair 2 provided an anomalously high score of 21 instead of 14.5 and 15.5 (See Figures S1 and S2). Due to carry over this resulted in continued maintenance of the elevated score by pair 2 in visit 4. In contrast, in case 5, pair 2 appeared to consistently score lower than pairs 1 and 3, through all ten visits and hence the overall slope for pair 2 is closely comparable to pairs 1 and 3 (Figure S1).

In aggregate, the data in Figures 3, suggest that it is probably desirable to have a clinical record assessed by 3-5 (rather than 2) students to flag occasional anomalies in scoring. Moreover we should also flag for greater scrutiny, records with (i) less than 5 visits disease curves and/or (ii) disease where cognition, memory, fine motor skills and speech are the prominent symptoms, since the students sometimes show a range of scores for these phenotypes. Despite these scoring anomalies, all seven disease severity curves mapped back with close correspondence to the progression described by Yanjanin et al. 2010 [6] (Figs. 3 C, D).

In summary the data show that both science-majors and non majors (but who were declared premed), can consistently be trained to assess and quantify NPC major and minor clinical disease symptoms and develop disease severity scales from clinical histories in a defined class structure.

### Scoring and disease severity curve generated from two new NPC patient records donated directly to our study

Two new clinical histories that were not previously assessed by Yanjanin et al. 2010 [6] were directly donated by families to our study in Spring 2010 and Spring 2011 respectively.

**Figure 4:**
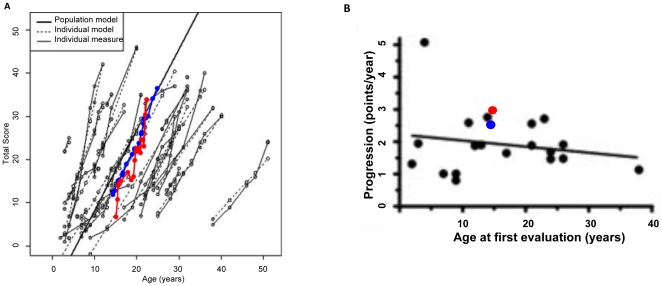
Assessment of two new NP-C patient medical records donated directly to the study. A. Disease severity curves of medical record donated in Spring 2010 (red) and Spring 2011 (blue). The Spring 2010 case was assessed by four students. The Spring 2011 case was assessed by 30 students**.** The data were overlaid on disease severity curves obtained by Yanjanin et al., 2010. B. The progression slopes of two curves shown in panel A were determined by linear regression and overlaid onto the progression slope created by Yanjanin et al. 2010.

In Spring 2010, a group of four students assessed the record, and progression of severity of disease (Figure S3) was mapped back to the cohort group of Yanjanin et al., 2010 [6] (Fig. 4A–B). Notably the linear regression yielded a slope of 2.676, (Fig. S3), which was greater than three standard deviations from the mean slope originally described by Yanjanin et al., 2010 [6] 1.9±0.2. This appeared to be due to the fact that patient displayed accelerated rapid disease progression in the last two clinical visits recorded. For the remaining data points, the curve and progression slope appeared to be in range (∼2.2) with the other patients previously reported by Yanjanin et al. 2010 [6].

The second case directly donated in Spring 2011 was assessed by all 30 students in class because in addition to generating the disease severity curve, we were also interested in investigating the inherent variation across individual students in a larger class. Thus, we had each of the thirty students assess the new record. The average disease severity curve was mapped back to the cohort of Yanjanin et al. 2010 [6] (Fig. 4A–B). The linear regression curve yielded a slope of 2.45, again suggesting accelerated disease progression compared to the cohort.

We therefore examined in detail the individual disease progression curves and scores that were derived from across clinical visits (in Figures S4 and S5). In aggregate these data showed that approximately 3–4 (∼10%) of 30 students had consistently lower scorers, with absolute scores that were as much as 2 standard deviations away from the mode (Fig. S5). However this bias did not change the slope, suggesting that the accelerated disease progression portrayed was consistently observed across operators. This was similar to the accelerated rapid disease progression in the last two clinical visits recorded in the first donated clinical record shown in Fig. 4A

### Creation of a clinical summary sheet

For each clinical case, students also created one to two page summary sheet displaying a history of medical visits and lab tests, onset of major and minors systems, utilization of drugs, known contraindication, allergies, hospitalizations, surgical procedures, all physicians and specialists previously visited including reason for visit, and herbal supplements. This summary provides a condensed insight for physicians treating NPC patients and is a service product that could be helpful to families.

## Discussion

Our study suggests that college students were able to review and assess clinical records once they achieved understanding of clinical NPC disease manifestation, diagnosis and management and learned the requisite medical terminology indicated and method of scoring. One difficulty was variability and sometimes limited information in individual medical histories. The method is dependent on the quality of existing medical records. The records from the NIH are equivalent to those from a university outpatient clinical setting, with evaluation by neurologists familiar with metabolic disorders. Records from primary care centers were also used, although the charts from standard hospital chart-systems, were relatively well organized since the specialist was integrated into the same system.

*In-class* discussions also focused on ambiguity of scoring domains and how to score inconsistently recorded data. After students independently graphed the plots, it was apparent that inter-individual variability based on ambiguity of recognizing domains were small and that the scoring process was robust. Discussion with clinical experts Forbes D. Porter M.D., Ph. D. and Nicole Yanjanin N.P. provided guidelines (previously reported in Yanjanin et al. 2010 [6]) on handling inconsistently recorded data. In early phases of the course, direct interaction with clinical practitioners greatly facilitated confidence in utilization and interpretation of scoring methods and data, although the 2011 class was able to complete finals scores prior to their interaction with clinical practitioners.

The take-home assessment of a single patient history was completed within 6 hours of out-of-class time. Assigning a record to three to four students per group would be sufficient to ensure accurate and verifiable assessment. Moreover once the group is trained (in the first four weeks), additional records could be assessed at the rate of one per week per student pair, for the remaining eight weeks. Finally the class size is easily scalable up to 50 students, suggesting significant, future, potential for assessing ∼200 patient records in the course of one semester. Thus this approach as the ability to produce a robust database from which the Natural History of a rare disease could be extracted. The limiting factor is obtaining sufficient number of records and thus coordination with a parent support organization (PSO) would be critical. PSOs may also be helpful to prospective studies, by providing attending physicians with a list of questions on clinical history and symptoms that serve as a checklist for the disorder, but mechanisms to ensure that such a list is systematically used, are also needed.

Assessment of the two medical records directly contributed to our study (received in Spring 2010 and 2011) supported the model of Yanjanin et al. (2010; 6) but also revealed, unexpected and accelerated disease progression, especially at later stages, not predicted by the severity scale of Yanjanin et al., 2010 [6]. One possible explanation for this is that the current scale is based on patients who can travel to the NIH, because travel for patients in wheel chairs, needing oxygen and other critical assistance is difficult. Additionally, travel to a tertiary clinical center typically requires travel with family members and a natural history study on late stage disease would need to be sustained over many more years, further complicating logistics and increasing costs to the point that it would be difficult to fund. This is in contrast to relatively low costs of retrospective analysis of a decade or more of existing medical records, by taking advantage of existing university infrastructure and training. The Yanjanin et al study continues to provide a reference point for the linear disease progression model, however additional data is needed to ascertain the relative contribution of quadratic disease progression (Table S2) and accurately model the full disease course.

Additional review of community-based assessment of patient records will reveal whether it can provide specific insight into the natural history at more severe stages of disease, in addition to accelerate the volume of assessments needed to strengthen disease scores to assess urgently needed, emerging therapies for NPC. It will also be of interest to determine whether community based assessment of patient records in other rare diseases can be undertaken to yield insights into disease processes and prognosis.

## Supporting Information

Figure S1 **Disease severity scores generated by pairs of students for cases 5-7 for curves shown in** **Figure 3B** **.**
(TIF)

Figure S2 **Disease severity scores generated by individual students for case 7.** Pair 1 Score, Pair 2 Score, and Pair 3 Score indicate student pairs and individual domain scores for a pair is separated by a comma, as shown.
(TIF)

Figure S3 **Disease severity curve for new case donated in Spring 2010.**
(TIF)

Figure S4 **Individual disease severity curves for new case donated in Spring 2011, as assessed by 30 individual students.**
(TIF)

Figure S5 **Individual disease severity scores for new case donated in Spring 2011, as assessed by 30 individual students.**
(TIF)

Table S1 (PDF)

Table S2 **Average linear and quadratic R^2^ values for disease severity curves associated with cases 1-7 (numbered as described in** **Figure 3B** **).** The suffix (Y) indicates that they were obtained from medical records reported in Yanjanin et al (2010). New cases donated directly to our study in 2010 and 2011, not previously reported by Yanjanin et al (2010) and assessed in Figure 4, are also indicated. *Log fit is best fit.
(DOCX)
