# Correction: Defining Natural History: Assessment of the Ability of College Students to Aid in Characterizing Clinical Progression of Niemann-Pick Disease, Type C

**DOI:** 10.1371/annotation/642295d5-8e46-467d-a9f9-2c6ee94116a9

**Published:** 2011-11-01

**Authors:** Jenny Shin, Katrina Epperson, Nicole M. Yanjanin, Jennifer Albus, Laura Borgenheimer, Natalie Bott, Erin Brennan, Daniel Castellanos, Melissa Cheng, Michael Clark, Margaret Devany, Courtney Ensslin, Nina Farivari, Shanik Fernando, Lauren Gabriel, Rani Gallardo, Moriah Castleman, Olimpia Gutierrez, Allison Herschel, Sarah Hodge, Anne Horst, Mary Howard, Evan James, Lindsey Jones, Mary Kearns, Mary Kelly, Christine Kim, Kinzie Kiser, Gregory Klazura, Chris Knoedler, Emily Kolbus, Lauren Lange, Joan Lee, Eileena Li, Wei Lu, Andrew Luttrell, Emily Ly, Katherine McKeough, Brianna McSorley, Catherine Miller, Sean Mitchell, Abbey Moon, Kevin Moser, Shane O'Brien, Paula Olivieri, Aaron Patzwahl, Marie Pereira, Craig Pymento, Erin Ramelb, Bryce Ramos, Teresa Raya, Stephen Riney, Geoff Roberts, Mark Robertshaw, Frannie Rudolf, Samuel Rund, Stephanie Sansone, Lindsay Schwartz, Ryan Shay, Edwin Siu, Timothy Spear, Catherine Tan, Marisa Truong, Mairaj Uddin, Jennifer VanTrieste, Omar Veloz, Elizabeth White, Forbes D. Porter, Kasturi Haldar

The third affiliation should be: "NPC Consortium for Community-Based Assessment of Patient Records, Notre Dame, Indiana." 

