# Supplementary figures and images for: Defining Natural History: Assessment of the Ability of College Students to Aid in Characterizing Clinical Progression of Niemann-Pick Disease, Type C
Source: PLoS One. 2011 Oct 3;6(10):e23666. doi: 10.1371/journal.pone.0023666 (PMC3184943; doi:10.1371/journal.pone.0023666)

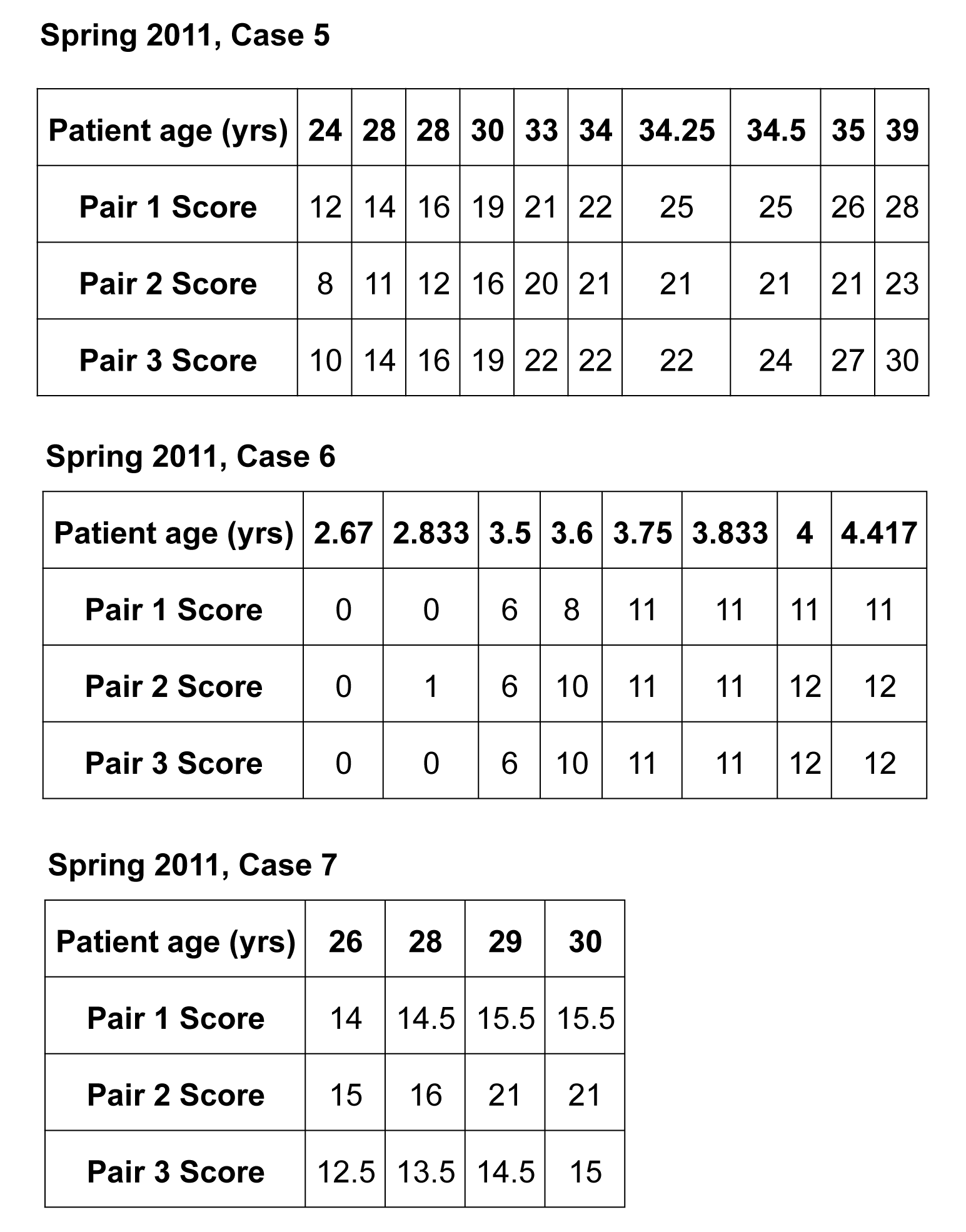

Supplement: Figure S1 — Disease severity scores generated by pairs of students for cases 5-7 for curves shown in Figure 3B . (TIF) [file pone.0023666.s001.tif]

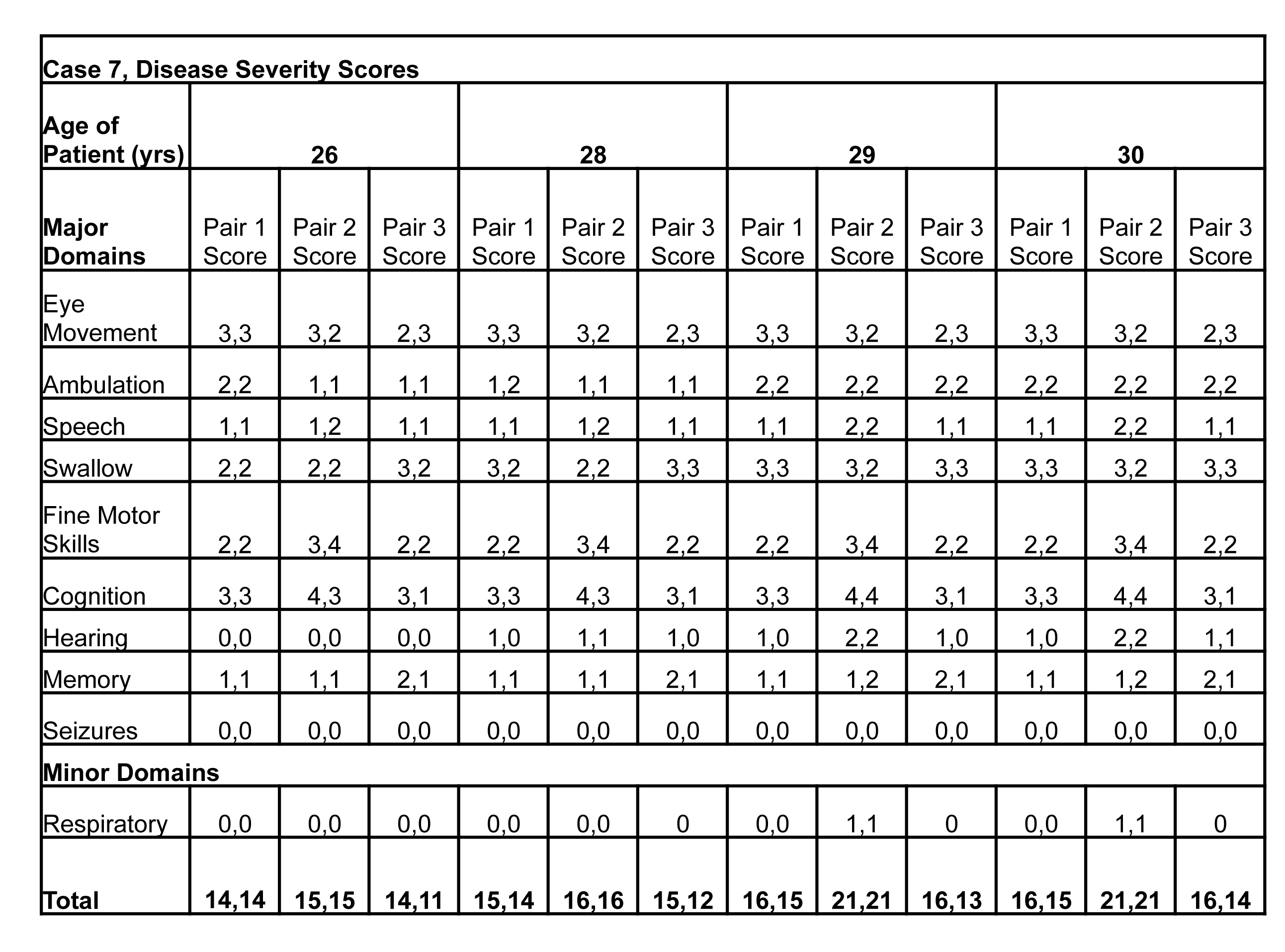

Supplement: Figure S2 — Disease severity scores generated by individual students for case 7. Pair 1 Score, Pair 2 Score, and Pair 3 Score indicate student pairs and individual domain scores for a pair is separated by a comma, as shown. (TIF) [file pone.0023666.s002.tif]

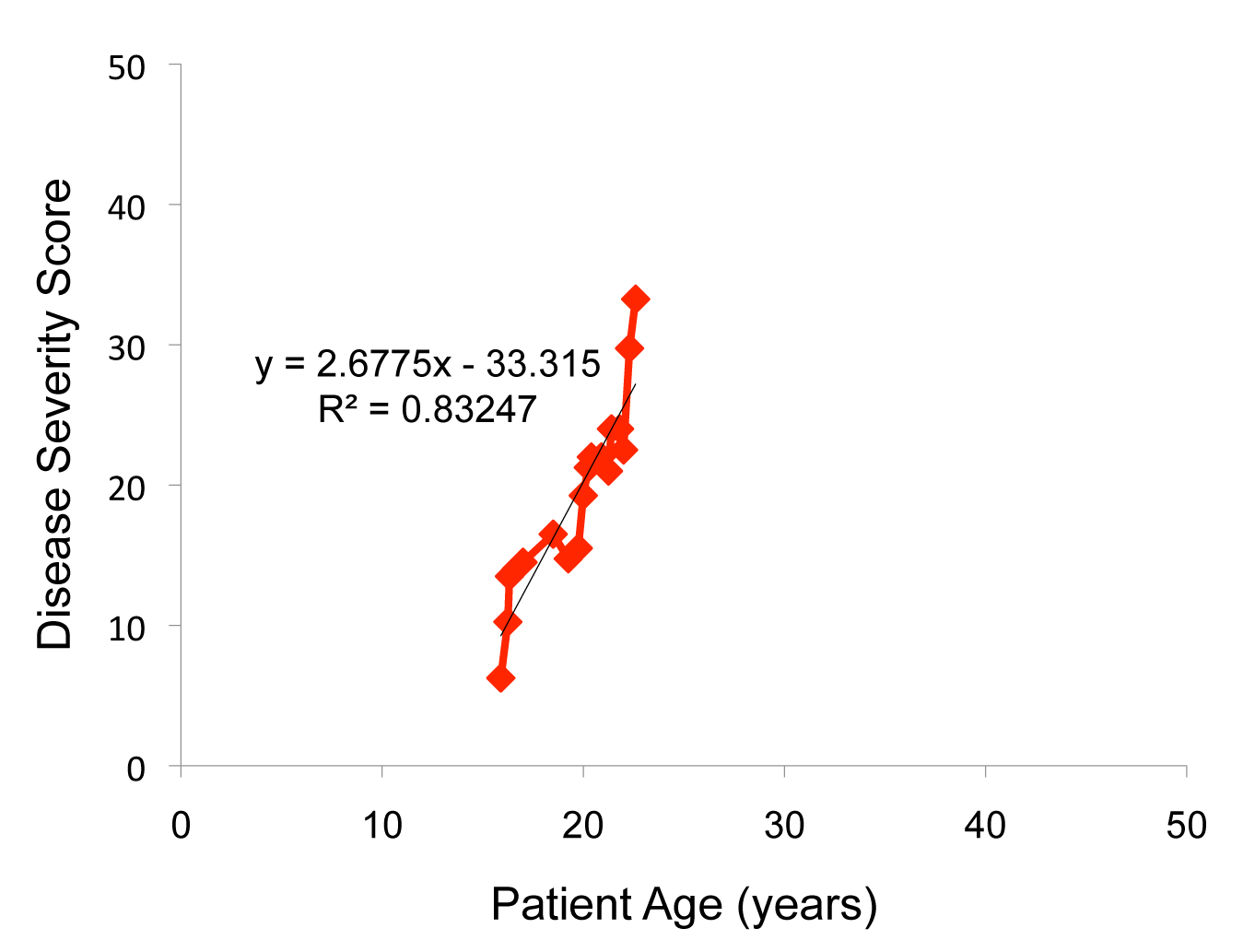

Supplement: Figure S3 — Disease severity curve for new case donated in Spring 2010. (TIF) [file pone.0023666.s003.tif]

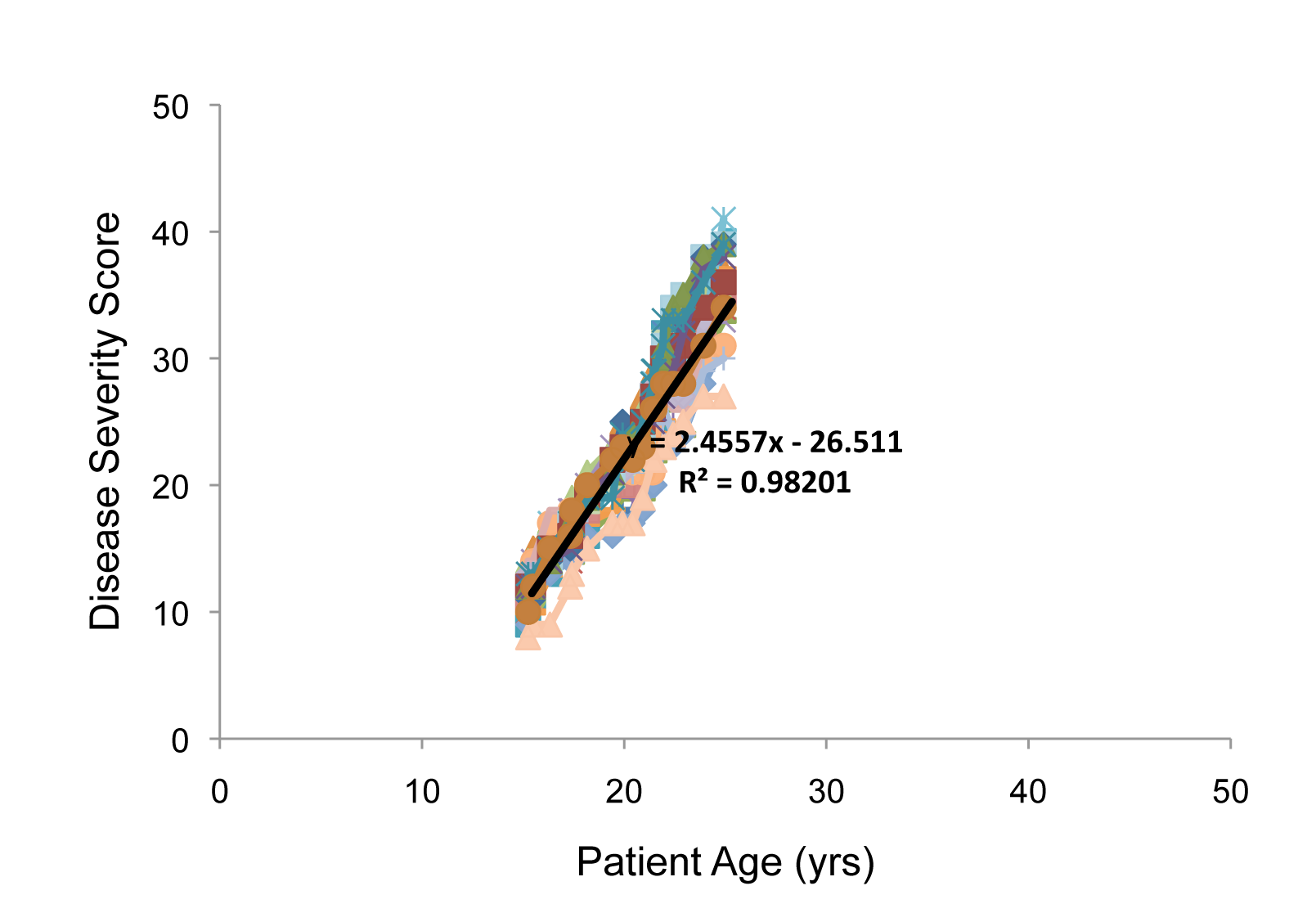

Supplement: Figure S4 — Individual disease severity curves for new case donated in Spring 2011, as assessed by 30 individual students. (TIF) [file pone.0023666.s004.tif]

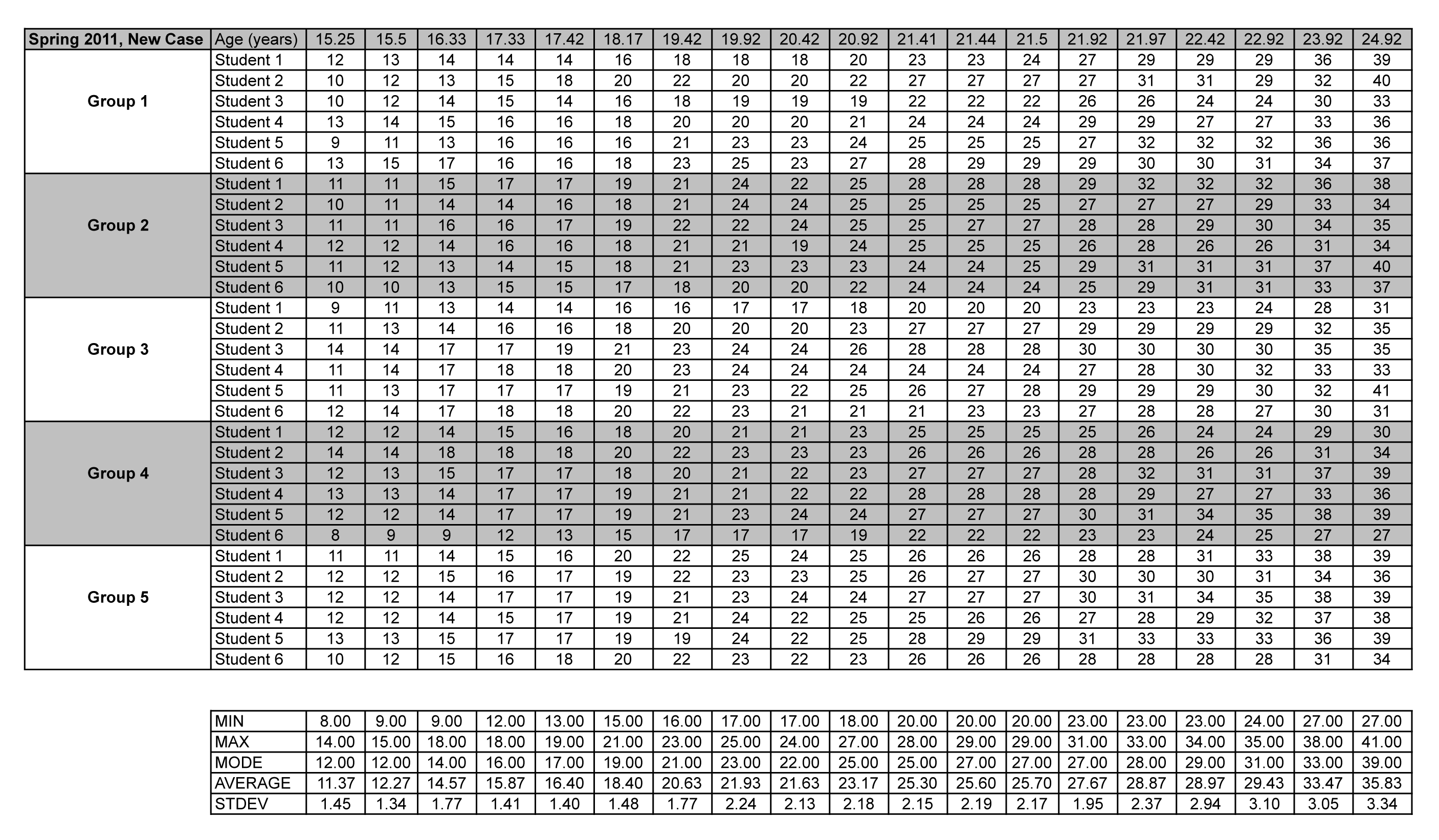

Supplement: Figure S5 — Individual disease severity scores for new case donated in Spring 2011, as assessed by 30 individual students. (TIF) [file pone.0023666.s005.tif]
