# Supplementary material for: Defining Natural History: Assessment of the Ability of College Students to Aid in Characterizing Clinical Progression of Niemann-Pick Disease, Type C
Source: PLoS One. 2011 Oct 3;6(10):e23666. doi: 10.1371/journal.pone.0023666 (PMC3184943; doi:10.1371/journal.pone.0023666)
Supplement: Table S1 — (PDF) [file pone.0023666.s006.pdf]

Supplementary Table 1

- Accommond amplitude: measurement of the eye's ability to focus clearly on objects at near distances
- Acoustic Immittance: measure of energy through the middle ear
- Adenectomy: surgical excision of a gland
- Adenoids- (or pharyngeal tonsils) a mass of lymphoid tissue situated at the very back of the nose, in the roof of the nasopharynx, where the nose blends into the mouth
- Adenoids: two masses of lymphoid tissue located at the back of the nose in the upper part of the throat that may obstruct normal breathing and make speech difficult when swollen
- Adenopathy- Enlarged lymph nodes which may be caused by local or generalized infection, inflammatory conditions, malignancy or more unusual disorders
- ADL: Activities of Daily Living, i.e.: eating, bathing, dressing, toileting, transferring, and mobility).
- Aeration- the process by which air is circulated through, mixed with or dissolved in a liquid or substance
- Albumin: A class of simple, water-soluble proteins that can be coagulated by heat and precipitated by strong acids and are found in egg white, blood serum, milk, and many other animal and plant juices and tissues
- Anisocytosis- a medical term meaning that a patient's red blood cells are of unequal size. This is found in anemia and other blood conditions
- Apgar Score- a simple and repeatable method to quickly and summarily assess the health of newborn children immediately after childbirth
- AST: aspartate aminotransferase . Low levels of AST are normally found in the blood. When body tissue or an organ such as the heart or liver is diseased or damaged, additional AST is released into the bloodstream.
- Aural fullness: feeling of air pressure build up in the ear
- Auscultation- the technical term for listening to the internal sounds of the body, usually using a stethoscope
- Auscultation: the act of listening, either directly or through a stethoscope or other instrument, to sounds within the body as a method of diagnosis
- Barognosia: ability to appreciate the weight of objects, or to differentiate objects of different weights.
- Bilateral Otitis media: ear infection in both ears
- Bilirubin: (A reddish-yellow bile pigment, derived from the degradation of heme. in the blood.)
- Bilirubin: a reddish bile pigment, $C_{33}H_{36}O_6N_4$, resulting from the degradation of heme by reticuloendothelial cells in the liver: a high level in the blood produces the yellow skin symptomatic of jaundice
- Celiac: digestive disease that damages the small intestine and interferes with absorption of nutrients from food. People who have celiac disease cannot tolerate gluten
- Clinodactyly- a medical term describing a bend or curvature of the fifth fingers (the "little fingers") toward the adjacent fourth fingers
- Clubbing: A condition in which the ends of the fingers and toes are enlarged and the nails are shiny and abnormally curved.
- Consanguinity- the property of being from the same lineage as another person
- Convergence amplitude: angle that eyes can move towards each other
- Corpuscular- A measure of the red blood cell volume
- Costal margin: sometimes referred to as the *costal arch*, is the medial margin formed by the false ribs -- specifically, from the seventh rib to the tenth rib.
- Cranial Nerves 2-12:

1st. Olfactory.	7th. Facial.
2d. Optic.	8th. Acoustic.
3d. Oculomotor.	9th. Glossopharyngeal.
4th. Trochlear.	10th. Vagus.
5th. Trigeminal.	11th. Accessory.

6th. Abducent. 12th. Hypoglossal.

- Creatine: an amino acid, $C_4H_9N_3O_2$, that is a constituent of the muscles of vertebrates and is phosphorylated to store energy used for muscular contraction
- Cyanosis- a blue coloration of the skin and mucous membranes due to the presence of > 5g/dl deoxygenated hemoglobin in blood vessels near the skin surface
- Cyanosis: blueness or lividness of the skin, as from imperfectly oxygenated blood
- Cytomegalovirus- a viral genus of the Herpesviruses group. HCMV infections are frequently associated with salivary glands, though they may be found throughout the body. HCMV infection can also be life threatening for patients who are immunocompromised. HCMV is also the virus most frequently transmitted to a developing fetus.
- Cytomegalovirus: common herpes-type virus that can cause fever, chills, sore throat, swollen glands, body aches, and fatigue
- Divergence amplitude: angle that eyes can move part from each other
- Dyslipidemia: disruption in the amount of lipids in the blood.
- Dystonia: Dystonia is a movement disorder, which causes involuntary contractions of your muscles. These contractions result in twisting and repetitive movements. Sometimes they are painful. Dystonia can affect just one muscle, a group of muscles or all of your muscles. Symptoms can include tremors, voice problems or a dragging foot.
- Eczema: inflammation of the epidermis
- Edema- an abnormal accumulation of fluid beneath the skin, or in one or more cavities of the body
- Edema: effusion of serous fluid into the interstices of cells in tissue spaces or into body cavities
- End intention dysmetria – Involuntary motions and the end of a voluntary movement
- Eosinophilia: Having a high concentration of eosinophills in the blood
- Epstein-Barr virus: can cause mononucleosis (mono) and is associated with some types of cancer, such as Burkitt's lymphoma and cancers of the mouth
- Etilogy- possible misspelling of etiology, or the study of the causes of diseases or pathologies
- Eustachian tube dysfunction – when the ear fails to maintain proper air pressure.
- Exanthem: wide spread rash
- F/U – follow up
- Fasciculations: muscular twitching involving the simultaneous contraction of contiguous groups of muscle fibers
- Fibrinogen- a soluble plasma glycoprotein that is synthesised by the liver, makes up fibrin (a fibrous protein involved in the clotting of blood)
- Filtrum- also known as the infranasal depression is the vertical groove in the upper lip
- Forced ductions: a maneuver to determine whether a mechanical obstruction is present in the eye; with forceps grasping an eye muscle, an attempt is made to passively move the eyeball in the direction of restricted rotation.
- Forced generations: attempting to move the eye mechanically – a "normal" eye will resist being pushed in one direction or another
- Gallop: A disordered rhythm of the heart characterized by three or four distinct heart sounds in each cycle and resembling the sound of a galloping horse
- Gauchers: most common of the lysosomal storage diseases. It is caused by a deficiency of the enzyme glucocerebrosidase (also known as acid β-glucosidase), leading to an accumulation of its substrate, the fatty substance glucocerebroside (also known as glucosylceramide).
- Gemfibrozil: Gemfibrozil is in a class of lipid-regulating medications called fibrates. It works be reducing the production of triglycerides in the liver. Gemfibrozil is used with diet changes (restriction of cholesterol and fat intake) to reduce the amount of cholesterol and triglycerides (other fatty substances) in the blood
- Gesell (pictures, child IDed them)
- Graphesthesia: the ability to recognize writing on the skin purely by the sensation of touch.
- Gravida II- a woman who is pregnant for the second time
- Hamartoma- a benign,[2] focal malformation that resembles a neoplasm in the tissue of its origin. This is not a malignant tumor, and it grows at the same rate as the surrounding tissues. It is

composed of tissue elements normally found at that site, but which are growing in a disorganized mass

- Hematocrit: the ratio of the volume of red blood cells to a given volume of blood so centrifuged, expressed as a percentage.
- Hepatosplenomegaly: Hepatomegaly is swelling of the liver beyond its normal size. The lower edge of the liver normally comes just to the lower edge of the ribs (costal margin) on the right side. In its normal state, the edge of the liver is thin and firm, and it cannot be felt with the fingertips below the edge of the ribs. It may be considered enlarged if a doctor can feel it in this area. If both the liver and spleen are enlarged, it is called hepatosplenomegaly.
- Histrelin: suppresses puberty by reducing the amount of hormones in the blood; used to treat central precocious puberty (CPP), a condition in which puberty begins at an unusually early age.
- Hormones
    - Follicle-stimulating hormone (FSH): a hormone synthesized and secreted by gonadotropes in the anterior pituitary gland. FSH regulates the development, growth, pubertal maturation, and reproductive processes of the human body.
    - Luteinizing hormone (LH): a hormone produced by the anterior pituitary gland that triggers ovulation in females.
    - DHEA sulfate: a weak male hormone (androgen) produced by the adrenal gland in both men and women. The adrenal gland is one of the major sources of androgens in women (the other being the ovaries, which produce testosterone). DHEA-sulfate is measured in women showing symptoms of male body characteristics (virilism) or excessive hair growth (hirsutism). It is also done in children who are maturing too early (precocious puberty).
- HVF: (Humphrey Visual Field) Measures field of view (“the flashing dot” test at the Optometrist)
- Hyperbilirubinemia: Excessive amounts of bilirubin (A reddish-yellow bile pigment, derived from the degradation of heme._ in the blood.) A severe, prolonged physiologic jaundice.
- Hypercholesterolemia: condition characterized by very high levels of cholesterol in the blood.
- Hyperchromic: of, relating to, or characterized by an increase in light absorption, especially of ultraviolet light
- Hyperlipidemia (Type II-B)- Hyperlipidemia with decreased LDL-receptor and increased ApoB
- Hyperlipidemia Type IV: high cholesterol and high blood triglycerides
- Hyperlipidemia- an elevation of lipids (fats) in the bloodstream
- Hyperlipidemia: elevated levels of lipids in the blood plasma
- Hypopharynx- the lower floor of the pharynx
- Interstitial fibrosis: scarring (or fibrosis) in the interstitium (or tissue between the air sacs), causing the lungs to becomes stiff
- LDH- Lactate Dehydrogenase
- Lingual Tonsils- rounded masses of lymphatic tissue that cover the posterior region of the tongue
- Lipomas: a benign tumor composed of fatty tissue
- Lymphadenopathy- a term meaning "disease of the lymph nodes." It is, however, almost synonymously used with "swollen/enlarged lymph nodes".
- Lymphocytes- a type of white blood cell in the vertebrate immune system
- Macrocytosis: abnormally large red blood cells
- Maxillary (sinuses)- Bones that form the upper jaw
- MCH: mean corpuscular hemoglobin, amount of hemoglobin in an average red blood cell
- MCHC: mean corpuscular hemoglobin concentration, the concentration of hemoglobin in an average red blood cell
- MCV: mean corpuscular volume, shows the size of the red blood cells
- Mediastinum- a non-delineated group of structures in the thorax (chest), surrounded by loose connective tissue
- Microcytosis- a blood disorder characterized by the presence of microcytes (abnormally small red blood cells) in the blood; often associated with anemia
- Microcytosis: abnormally small red blood cells
- MMT: (manual muscle test) A test of muscle strength
- Monospot Screen- A screen for Mono

- MR spectroscopy: Additional procedure during an MRI scan which provides additional information on the biochemical processes and chemicals present in the body's cells
- Murmur: An abnormal sound, usually emanating from the heart, that sometimes indicates a diseased condition
- Nares- The nostrils or nasal passages
- Negative middle ear pressure: the pressure on the ear drum is greater on the outside then the inside (like in an airplane)
- Neoplasm- A structure resulting from the abnormal proliferation of cells
- Niemann Pick A/B: Insufficient activity of the enzyme acid sphingomyelinase causes the build up of toxic amounts of sphingomyelin, a fatty substance present in every cell of the body.
- Normocephalic- a person whose head and all major organs of the head are in a normal condition and without significant abnormalities
- Nystagmus- a form of involuntary eye movement. It is characterized by alternating smooth pursuit in one direction and saccadic movement in the other direction
- Nystagmus: Involuntary rhythmic oscillation of the eyeballs, either pendular or with a slow and fast component.
- Nystagmus: involuntary usually rapid movement of the eyeballs (as from side to side) occurring normally with dizziness during and after \bodily rotation or abnormally following head injury or as a symptom of disease
- Ocular Oscillations: back and forth movements of eyes.
- Organomegaly- the abnormal enlargement of organs
- Organomegaly: Abnormal enlargement of the viscera
- Otitis Media- is inflammation of the middle ear, or middle ear infection
- Otitis Media: an ear infection
- Ovalocytes: aberrant shaped red blood cells
- Palpated- used as part of a physical examination in which an object is felt to determine its size, shape, firmness, or location
- Papilloma- A small benign epithelial tumor, such as a wart, consisting of an overgrowth of cells on a core of smooth connective tissue
- Papillomatous: a benign tumor of the skin or mucous membrane consisting of hypertrophied epithelial tissue, as a wart
- Papules- a circumscribed, solid elevation of skin with no visible fluid, varying in size from a pinhead to 1cm
- Para I- Live births that a mother has had
- Percussion- Used to assess the amount of air in a lung
- Percussion: the striking or tapping of the surface of a part of the body for diagnostic or therapeutic purposes
- Percutaneous- any medical procedure where access to inner organs or other tissue is done via needle-puncture of the skin
- Phosphatase: Any of numerous enzymes that catalyze the hydrolysis of esters of phosphoric acid and are important in the absorption and metabolism of carbohydrates, nucleotides, and phospholipids and in the calcification of bone
- Phospholipidosis- a lipid storage disorder in which excess phospholipids accumulate within cells
- Phospholipidosis:
- Polychromasia: variation in the hemoglobin content of erythrocytes.
- Polys- polys or PMN's - polymorphonuclear leukocytes
- PRN: *pro re nata (“When necessary”)*
- Prothrombin- A glycoprotein formed by and stored in the liver and present in the blood plasma that is converted to thrombin in the presence of thromboplastin and calcium ion during blood clotting
- Protuberant (abdomen)- thrusting out from a surrounding or adjacent surface often as a rounded mass
- Pure tone Auditory: is the testing of hearing ability by playing pure tones and determining the lowest dB that a person can hear
- RAST: Form of ex vivo allergy test where amount of patient IgE that reacts with allergen is measured.

- Resection: partial or complete removal of an organ or other bodily structure
- Respiratory syncytial virus (RSV): respiratory virus that infects the lungs and breathing passages.
- Reticulocyte count: measures how fast red blood cells called reticulocytes are made by the bone marrow and released into the blood
- S. agalactiae: species of the normal flora of the female urogenital tract and rectum. Its chief clinical importance is that it can be transferred to a neonate passing through the birth canal and can cause serious group B streptococcal infection. In the western world, S. agalactiae is a major cause of bacterial septicemia of the newborn, which can lead to death or long-term sequelae such as hearing loss
- Saccades: a small rapid jerky movement of the eye especially as it jumps from fixation on one point to another (as in reading)
- Sedimentation rate:
- Sedimentation rate: measures how quickly red blood cells (erythrocytes) settle in a test tube in one hour;
- Serous effusion bilaterally: collection of non-infected fluid in the middle ear space that may accumulate in the middle ear as a result of a cold, sore throat or upper respiratory infection
- Serum DHEA: levels indicate adrenal activity
- SGOT- Serum glutamic oxaloacetic transaminase, an enzyme that is normally present in liver and heart cells. SGOT is released into blood when the liver or heart is damaged.
- SGPT- Serum glutamic pyruvic transaminase, an enzyme that is normally present in liver and heart cells. SGPT is released into blood when the liver or heart is damaged.
- Shistocytes: fragmented red blood cells
- Smith Test
- Sphingomyelinase- a hydrolase enzyme that is involved in sphingolipid metabolism reactions. SMase is a member of the DNase I super family of enzymes and is responsible for breaking sphingomyelin (SM) down into phosphocholine and ceramide (a family of lipid molecules, functions as a cellular signal including the regulation of differentiation, proliferation, programmed cell death (PCD), and apoptosis of cells). The activation of SMase has been suggested as a major route for the production of ceramide in response to cellular stresses.
- Sphingomyelinase: an enzyme acid, which is needed to break down sphingomyelin, a fatty substance found mostly in the brain and nervous system, deficiency results in abnormal accumulation of excessive amounts of sphingomyelin in many organs of the body such as the liver, spleen, and brain.
- Stridor: a high-pitched sound resulting from turbulent airflow in the upper airway.
- Supraglottic- The part of the larynx (air pathway) above the glottis (where the vocal cords are located)
- Supraglottic: situated or occurring above the glottis
- Synagis (Palivizumab) : Monoclonal antibody against RSV
- Tangier's Disease- a rare inherited disorder characterized by a severe reduction in the amount of high density lipoprotein (HDL)
- Tanner: stages of physical development in children, adolescents and adults. The stages define physical measurements of development based on external primary and secondary sex characteristics,
- Tear drop cells: abnormal erythrocytes have a teardrop, or pear like, shape. They are associated with disorders with an abnormal spleen
- Thrill: The vibration accompanying a cardiac or vascular murmur, detectible on palpation
- Thrombin- a coagulation protein that has many effects in the coagulation cascade
- Thromboplastin- A protease that converts prothrombin to thrombin in the early stages of blood clotting
- Tight heal cords – tightness of the heal tendon. Seen in neurological conditions in addition to congenital birth defects.
- Titer- a measure of concentration
- Titubations- The staggering or stumbling gait characteristic of certain nervous disorders
- Titubations: The staggering or stumbling gait characteristic of certain nervous disorders
- Tomography- imaging by sections or sectioning

- Tonsillar and adenoidal hypertrophy: abnormal enlargement or excessive growth of tonsils and adenoids
- Tonsillectomy: surgical removal of the tonsils
- Tracheomalacia- a condition characterized by flaccidity of the tracheal support cartilage which leads to tracheal collapse especially when increased airflow is demanded
- Tracheomalacia: condition where the walls of the trachea are blocked or collapse because of weak or soft tissue
- Transferrin- a blood plasma protein for iron ion delivery
- Tympanic Membranes- a thin membrane that separates the external ear from the middle ear. Its function is to transmit sound from the air to the ossicles inside the middle ear
- UE ROM: Upper extremity range of motion
- Vallecula: an anatomical groove, channel, or depression (ex: groove between the base of the tongue and the epiglottis)
- Verricuform: shaped like a wart or warts
- Verruciform Xanthoma- a papilloma look-alike lesion which seems not to be associated with HPV but is perhaps a response to local trauma
- Viscera: the organs in the cavities of the body, especially those in the abdominal cavity
- Waldeyer's Ring- an anatomical term describing the lymphoid tissue ring located in the pharynx and to the back of the oral cavity
- WNL: With in normal limits
- Xanthoma: Xanthoma is a skin condition in which fat builds up under the surface of the skin. Xanthomas are common, particularly among older adults and persons with high blood lipids.
- Xanthomas: lesions characterized by accumulations of lipid-laden macrophages. Xanthomas can be a reflection of lipid metabolism alteration or a result of local cell dysfunction.
- Xanthomas: visible fatty deposits under the skin
