# Supplementary material for: Defining Natural History: Assessment of the Ability of College Students to Aid in Characterizing Clinical Progression of Niemann-Pick Disease, Type C
Source: PLoS One. 2011 Oct 3;6(10):e23666. doi: 10.1371/journal.pone.0023666 (PMC3184943; doi:10.1371/journal.pone.0023666)
Supplement: Table S2 — Average linear and quadratic R2 values for disease severity curves associated with cases 1-7 (numbered as described in Figure 3B ). The suffix (Y) indicates that they were obtained from medical records reported in Yanjanin et al (2010). New cases donated directly to our study in 2010 and 2011, not previously reported by Yanjanin et al (2010) and assessed in Figure 4, are also indicated. *Log fit is best fit. (DOCX) [file pone.0023666.s007.docx]

| **CASE** | **LINEAR R^2^ VALUE** | **QUADRATIC R^2^ VALUE** |
| --- | --- | --- |
| 1 (Y) | 0.9755 | 0.96538 |
| 2 (Y) | 0.86241 | 0.99154 |
| 3 (Y) | 0.82989 | 0.90787* |
| 4 (Y) | 0.94231 | 0.96516 |
| 5 (Y) | 0.95208 | 0.89613 |
| 6 (Y) | 0.98098 | 0.97254 |
| 7 (Y) | 0.96571 | 0.95775 |
| NEW 2010 CASE | 0.8325 | 0.98526 |
| NEW 2011 CASE | 0.98201 | 0.8654 |

**Supplementary Table 2**

**Supplementary Table 2:**  Average linear and quadratic R^2^ values for disease severity curves associated with cases 1-7 (numbered as described in Figure 3B). The suffix (Y) indicates that they were obtained from medical records reported in Yanjanin et al (2010). New cases donated directly to our study in 2010 and 2011, not previously reported by Yanjanin et al (2010) and assessed in Figure 4, are also indicated. *Log fit is best fit.
